# Obesity alters the *in vivo* mechanical response and biochemical properties of cartilage as measured by MRI

**DOI:** 10.1186/s13075-018-1727-4

**Published:** 2018-10-17

**Authors:** Amber T Collins, Micaela L Kulvaranon, Hattie C Cutcliffe, Gangadhar M Utturkar, Wyatt A R Smith, Charles E Spritzer, Farshid Guilak, Louis E DeFrate

**Affiliations:** 1 Department of Orthopaedic Surgery, Duke University, Box 3093, Duke University Medical Center, Durham, NC 27710 USA; 2 Department of Biomedical Engineering, Duke University, Campus Box 90281, 101 Science Drive, Durham, 27708 NC USA; 3 Department of Mechanical Engineering and Materials Science, Duke University, Campus Box 90300, Hudson Hall, Durham, 27708 NC USA; 4 Department of Radiology, Duke University, Box 3808, Duke University Medical Center, Durham, 27710 NC USA; 5 Department of Orthopaedic Surgery, Washington University and Shriners Hospitals for Children, Campus Box 8233, Couch Research Building, Room 3121, St. Louis, 63110 MO USA

**Keywords:** Obesity, Cartilage, Magnetic resonance imaging (MRI), Proteoglycan, mechanobiology, stress test

## Abstract

**Background:**

Obesity is a primary risk factor for the development of knee osteoarthritis (OA). However, there remains a lack of *in vivo* data on the influence of obesity on knee cartilage mechanics and composition. The purpose of this study was to determine the relationship between obesity and tibiofemoral cartilage properties.

**Methods:**

Magnetic resonance images (3T) of cartilage geometry (double-echo steady-state) and T1rho relaxation of the knee were obtained in healthy subjects with a normal (n = 8) or high (n = 7) body mass index (BMI) before and immediately after treadmill walking. Subjects had no history of lower limb injury or surgery. Bone and cartilage surfaces were segmented and three-dimensional models were created to measure cartilage thickness and strain. T1rho relaxation times were measured before exercise in both the tibial and femoral cartilage in order to characterize biochemical composition. Body fat composition was also measured.

**Results:**

Subjects with a high BMI exhibited significantly increased tibiofemoral cartilage strain and T1rho relaxation times (*P* <0.05). Tibial pre-exercise cartilage thickness was also affected by BMI (*P* <0.05). Correlational analyses revealed that pre-exercise tibial cartilage thickness decreased with increasing BMI (R^2^ = 0.43, *P* <0.01) and body fat percentage (R^2^ = 0.58, *P* <0.01). Tibial and femoral cartilage strain increased with increasing BMI (R^2^ = 0.45, *P* <0.01; R^2^ = 0.51, *P* <0.01, respectively) and increasing body fat percentage (R^2^ = 0.40, *P* <0.05; R^2^ = 0.38, *P* <0.05, respectively). Additionally, tibial T1rho was positively correlated with BMI (R^2^ = 0.39, *P* <0.05) and body fat percentage (R^2^ = 0.47, *P* <0.01).

**Conclusions:**

Strains and T1rho relaxation times in the tibiofemoral cartilage were increased in high BMI subjects compared with normal BMI subjects. Additionally, pre-exercise tibial cartilage thickness decreased with obesity. Reduced proteoglycan content may be indicative of pre-symptomatic osteoarthritic degeneration, resulting in reduced cartilage thickness and increased deformation of cartilage in response to loading.

## Background

Obesity is a major risk factor for osteoarthritis (OA) [1–3] and the incidence of knee OA in obese individuals is four times greater than that in healthy weight controls [4]. Whereas the association between OA and obesity has been established, the mechanisms by which obesity increases the risk for OA are not well understood. Some studies attribute the increased risk of OA with obesity to increased joint loading due to elevated body mass [5, 6]; however, more recently, it has been suggested that a combination of biomechanical and metabolic factors, such as cartilage catabolism due to adipokine-related inflammation, plays an important role in this relationship [7, 8]. Furthermore, the presence of OA in non-weight-bearing joints of obese subjects [9, 10] suggests that factors other than mechanical loading potentially contribute to disease progression.

Nonetheless, there remains a lack of *in vivo* data describing the effects of obesity on cartilage composition and mechanical function. Although gait analysis studies can provide estimates of the loads experienced by the knee joint [11–13], it is unclear how these estimated loads relate to local *in vivo* cartilage deformation. Cartilage is a biphasic viscoelastic material due to the time-dependent exudation of water that occurs following mechanical loading of the tissue. Owing to the low permeability of the cartilage matrix, recovery of water back into the matrix once unloaded is not immediate [14–17]. Several studies have used magnetic resonance imaging (MRI) to characterize the *in vivo* deformation of cartilage by taking advantage of this time-dependent biomechanical recovery following loading [14–19]. Thus, this technique can be used to assess the effects of obesity on *in vivo* cartilage mechanics.

Previously, Widmyer et al. used MRI to show that increased body mass index (BMI) is associated with increased diurnal cartilage strains in the knee when compared with normal weighted controls; diurnal strain was defined as change in cartilage thickness from morning to evening [18]. However, it is unclear whether these increased cartilage strains associated with obesity are due solely to greater body mass or alterations in cartilage composition or both. Currently, there are limited *in vivo* data quantifying how obesity relates to alterations in cartilage composition and how these changes are related to altered mechanical function. Quantitative MRI techniques, such as T1rho-weighted imaging, have been used to quantify *in vivo* proteoglycan content in cartilage [20–24] and therefore can be used to assess changes in cartilage composition with obesity. The objective of this study was to assess how obesity alters both the *in vivo* mechanical function and composition of cartilage. We hypothesized that obesity is associated with a reduction in proteoglycan content, as evidenced by increased T1rho relaxation times. Additionally, we hypothesized that these alterations in composition result in decreased cartilage stiffness, which will be reflected by increased *in vivo* cartilage strain in response to mechanical loading. Our overall hypothesis is that obese subjects exhibit “pre-OA” changes in both articular cartilage composition and mechanical function that precede the onset of symptomatic OA.

## Methods

### Subject recruitment

Following approval by the institutional review board of Duke University Medical Center, eight subjects (five males and three females; mean age 30 years, range 23–43; mean height 70 in., range 64–74) with a normal BMI (mean 22.2; range 18–25) and seven subjects (three males and four females; mean age 32, range 22–45; mean height 66 in., range 63–71) with a high BMI (mean 32.8; range 30–36), who were otherwise healthy, were recruited for participation in this study. The normal BMI and high BMI groups were statistically significantly different with regard to BMI (*P* <0.0001, *t* test). However, no statistically significant differences were detected with regard to age (*P* = 0.64, *t* test), height (*P* = 0.08, *t* test), or the distribution of males and females between groups (*P* = 0.613, Fisher’s exact test). Previous work from our lab investigating cartilage strains in healthy subjects tested eight subjects and found significant changes in cartilage thickness as a result of treadmill walking [15]. Therefore, we aimed to recruit and test a similar number of subjects per group in the present study. All subjects provided informed written consent before beginning the study. Subjects were excluded if they had a history of lower limb injury, surgery, or symptoms related to OA.

### Study procedure

In order to minimize the effect of diurnal cartilage loading [14, 18, 19], subjects were tested early in the morning and instructed not to perform any strenuous activities on the day prior to and the morning of testing. Upon arrival, subjects lay supine for 45 min prior to the pre-exercise MRI scan to allow their knee cartilage to relax to its baseline, unloaded state in a room adjacent to the MRI scanner [19]. Following this relaxation period, subjects were transported to the MRI scanner in a wheelchair. Pre-exercise MRI images of each subject’s right knee were taken in the sagittal plane using a 3.0 T MRI scanner with an eight-channel knee coil [25] (Trio Tim, Siemens Medical Solutions USA, Malvern, PA, USA). A three-dimensional (3D) double-echo steady-state (DESS) sequence—flip angle: 25°; echo time (TE): 6 ms; repetition time (TR): 17 ms; field of view (FOV): 16 × 16 cm; matrix: 512 × 512 pixels; resolution: 0.3 × 0.3 × 1.0 mm—was used to obtain anatomical images of the bones and articular cartilage, allowing for pre-exercise cartilage thickness measurements. A T1rho-weighted imaging sequence with a 3D fast imaging with steady-state precession (FISP) acquisition—flip angle: 15°; TE: 5.9 ms; TR: 3500 ms; FOV: 14 × 14 cm; matrix: 256 × 256 pixels; resolution: 1.1 × 0.5 × 3.0 mm; B1: 500 Hz; spin lock time (TSL): 5, 10, 40, 80 ms—was collected in order to assess proteoglycan content within the cartilage [14, 15]. Following the pre-exercise MRI scan, subjects were transported by wheelchair to an adjacent room where they walked on a treadmill for 20 min. Walking speed was normalized to the subject’s leg length using the Froude number (Fr) (Fr = v^2^/(L×g)) [26], which uses leg length (L) as measured from the greater trochanter of the femur to the ground surface, and the gravitational constant (g = 9.8 m/s^2^) in order to calculate a normalized walking speed (v). Subjects walked at a Froude number of 0.25, which corresponds to an adult walking at a comfortable pace [26]. Subjects also wore a pedometer to record the number of steps taken during their 20-min walk. Immediately following exercise, subjects were transported back to the MRI scanner for a post-exercise DESS sequence scan which was used to measure post-exercise cartilage thickness. Lastly, each subject’s body composition (weight and body fat percentage) was measured immediately following the post-exercise MRI scan by using a bioelectrical impedance scale (InBody230, BioSpace Inc., Cerritos, CA, USA) [27].

### Data analysis

**Fig. 1:**
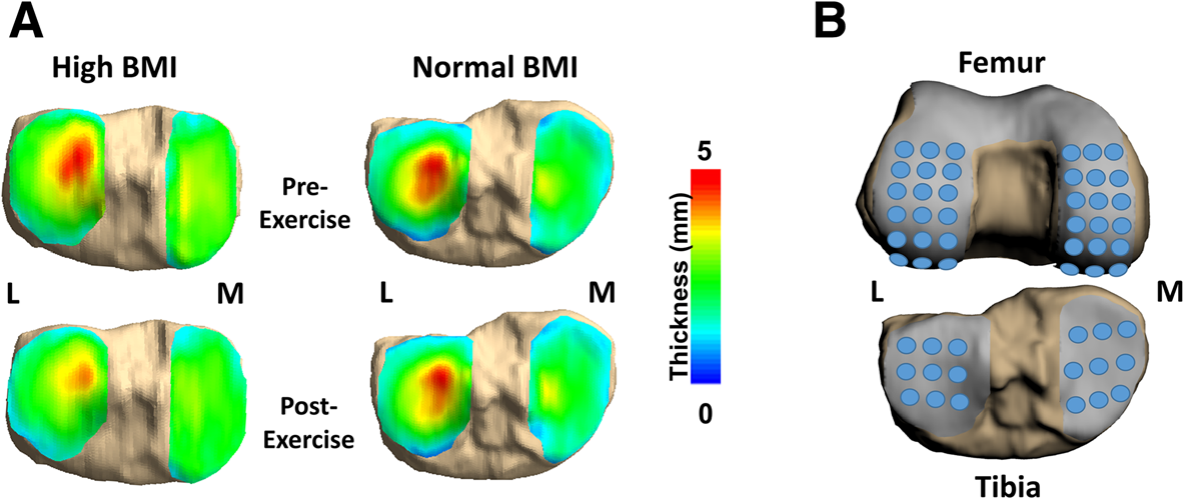
**a** Representative tibial cartilage thickness maps from a high body mass index (BMI) subject and a normal BMI subject. The color thickness maps demonstrate greater changes in the high BMI subject compared with the normal BMI subject following the 20-min walking task in both the medial (M) and lateral (L) aspects of the tibial cartilage. **b** Femur and tibia with articular cartilage surfaces demonstrating the grid point sampling locations. The tibial cartilage surfaces were sampled from 18 points, and the femoral cartilage surfaces were sampled from 36 points

The tibial and femoral bony and articular cartilage surfaces were segmented on the DESS images by using solid modeling software (Rhinoceros; Robert McNeel & Associates, Seattle, WA, USA) [14–16]. Segmentations from each DESS MRI slice were compiled to create 3D mesh models of the proximal tibia and distal femur as well as of both the associated articulating surfaces. The pre-exercise and post-exercise models were registered together by using an iterative closest-point algorithm, allowing for site-specific comparisons of cartilage thickness between the pre- and post-exercise scans (Geomagic Studio; Geomagic, 3D Systems, Valencia, CA, USA) [28]. Cartilage thickness maps were generated by calculating the distance from each vertex on the cartilage surface mesh to its nearest vertex on the corresponding bone surface mesh (Fig. 1a). Thickness measurements of both the tibial and femoral cartilage were averaged within uniformly spaced points, each with a radius of 2.5 mm. Eighteen points were placed on the tibial cartilage (9 points on each tibial plateau), and 36 points were placed on the femoral cartilage (18 points on each condyle) (Fig. 1b) [29]. Strain at each of these points was calculated as the difference between the pre- and post-exercise thickness, divided by the pre-exercise thickness [14]. These strains were averaged to generate mean cartilage strains representing strain across the tibial and femoral cartilage. The methodology used in the present study has been previously validated to measure cartilage thickness in the tibiofemoral joint to within a resolution of 1% [19, 29].

**Fig. 2:**
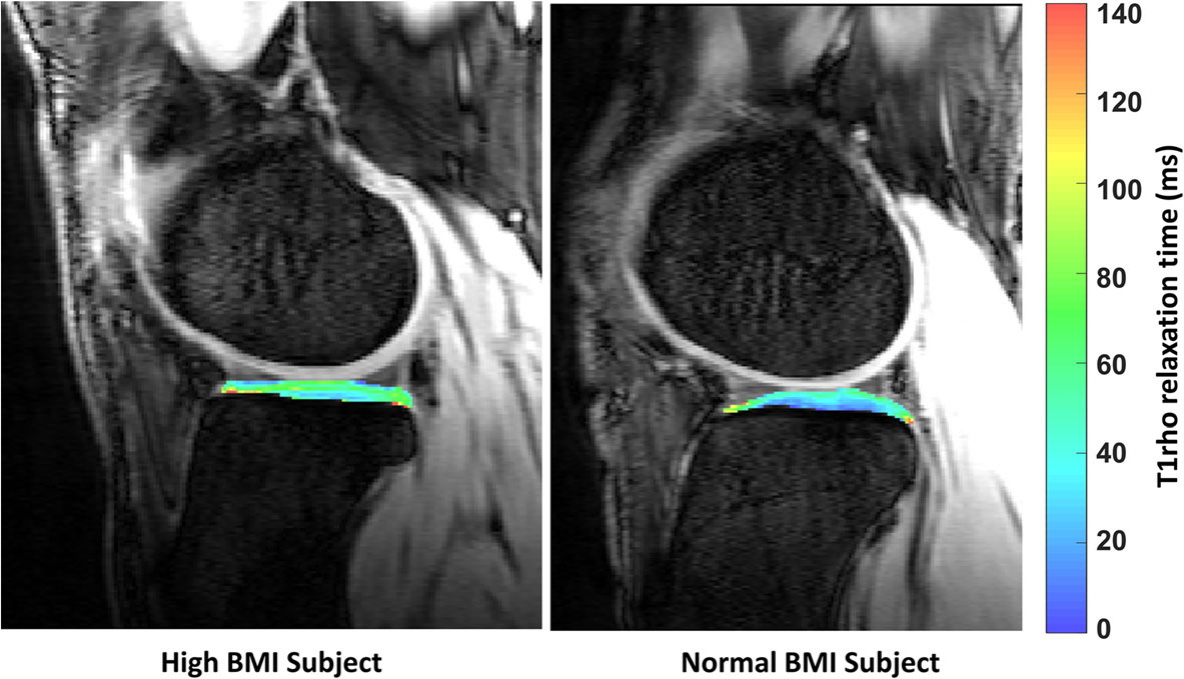
Color map of tibial cartilage T1rho relaxation times in one representative high body mass index (BMI) subject and one representative normal BMI subject

T1rho relaxation time maps were generated from the pre-exercise T1rho MRI images. The tibial and femoral cartilage were manually segmented from the TSL = 5 ms image (Fig. 2). For each voxel within the segmented cartilage regions, T1rho relaxation times were calculated by assessing the exponential decay of the MRI signal intensity with increasing spin lock time (TSL). This was done by fitting the following equation [30]:

$$ S(TSL)={S}_0{e}^{- TSL/{T}_{1\rho }}. $$

Here, S(TSL) represents signal intensity for a given voxel, S_0_ represents initial signal intensity, and TSL represents spin lock time. T1rho relaxation times of all voxels within the segmented region of the tibial cartilage were averaged, as were the relaxation times of all voxels of the femoral cartilage.

### T1rho repeatability analysis

The repeatability of this technique was assessed by comparing baseline tibial and femoral cartilage T1rho relaxation times acquired from four male test subjects (mean age 32, range 27–40; mean BMI 22.3, range 18–24) during two separate MRI sessions which occurred within a 2-week period. Subjects were tested at 8 am with a 45-min rest period prior to each testing session. The coefficient of variation of the acquired T1rho relaxation times was determined to be 1.4%.

### Statistical analysis

Two-way repeated measures analysis of variance (ANOVA) was performed to determine the influence of BMI (high versus normal) and location (femur versus tibia) on cartilage strain, T1rho relaxation time, and pre-exercise cartilage thickness. Fisher’s least significant difference (LSD) test was used for post hoc comparisons in cases where the ANOVA indicated a significant interaction between variables. Additionally, simple linear regressions were performed to analyze relationships between body composition (BMI and body fat percentage) and tibial and femoral cartilage properties (cartilage thickness, strain, and T1rho relaxation time). Statistical significance was defined as a *P* value of less than 0.05.

## Results

**Fig. 3:**
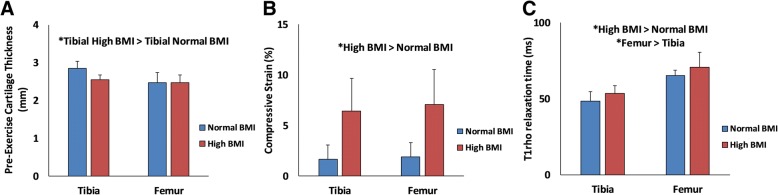
**a** Pre-exercise cartilage thickness **b** Cartilage compressive strain and **c** T1rho relaxation time. Data are presented as mean ± standard deviation. Asterisk indicates a significant effect. Abbreviation: *BMI* body mass index

Overall, high BMI subjects had significantly decreased resting tibial cartilage thickness (*P* <0.05, Fig. 3a) compared with normal BMI subjects. Additionally, high BMI subjects had increased compressive tibiofemoral cartilage strains compared with normal BMI subjects (*P* <0.01, Fig. 3b). Finally, high BMI subjects had elevated tibiofemoral T1rho relaxation times compared with normal BMI subjects (*P* = 0.03, Fig. 3c), and femoral T1rho relaxation times were greater than tibial T1rho relaxation times (*P* <0.01).

**Fig. 4:**
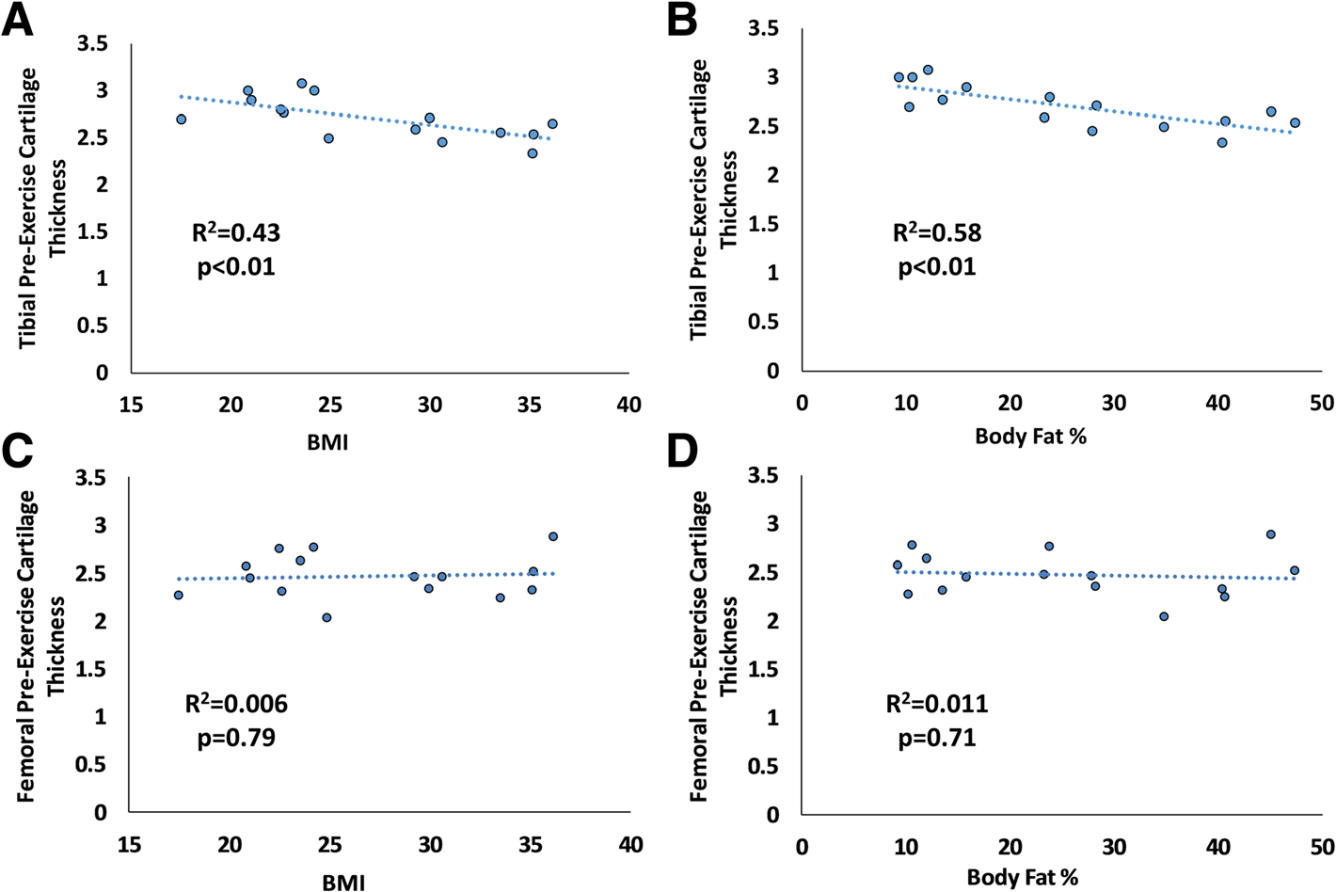
**a, b** Tibial cartilage pre-exercise thickness was significantly correlated with body mass index (BMI) (*P* <0.01) and body fat percentage (*P* <0.01). **c, d** Femoral cartilage pre-exercise thickness was not significantly correlated with BMI (*P* = 0.79) or body fat percentage (*P* = 0.71)

**Fig. 5:**
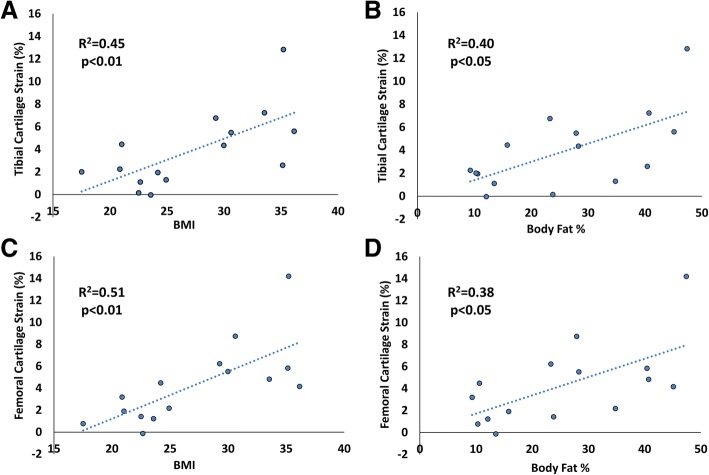
**a, b** Tibial cartilage compressive strain was significantly correlated with body mass index (BMI) (*P* <0.01) and body fat percentage (*P* <0.05). **c, d** Femoral cartilage strain was also significantly correlated with BMI (*P* <0.01) and body fat percentage (*P* <0.05)

As expected, BMI was significantly correlated with body fat percentage (*P* <0.05, R^2^ = 0.79). Additionally, tibial pre-exercise cartilage thickness was negatively correlated with BMI (*P* <0.01, R^2^ = 0.43, Fig. 4a), with 12% thinner cartilage in high BMI subjects compared with normal BMI subjects. Pre-exercise tibial cartilage thickness was also negatively correlated with body fat percentage (*P* <0.01, R^2^ = 0.58, Fig. 4b). Tibial cartilage strain was positively correlated with BMI (*P* <0.01, R^2^ = 0.45, Fig. 5a), with a nearly fourfold increase in high BMI subjects compared with normal BMI subjects. Tibial strain was also positively correlated with body fat percentage (*P* <0.05, R^2^ = 0.40, Fig. 5b).

Femoral pre-exercise cartilage thickness was not significantly correlated to either BMI (*P* = 0.79, R^2^ = 0.006) or body fat percentage (*P* = 0.71, R^2^ = 0.011). However, femoral cartilage strain was positively correlated with BMI (*P* <0.01, R^2^ = 0.51, Fig. 5c), again with a nearly fourfold increase in high BMI subjects compared with normal BMI subjects. Additionally, femoral strain was also positively correlated with body fat percentage (*P* <0.05, R^2^ = 0.38, Fig. 5d).

**Fig. 6:**
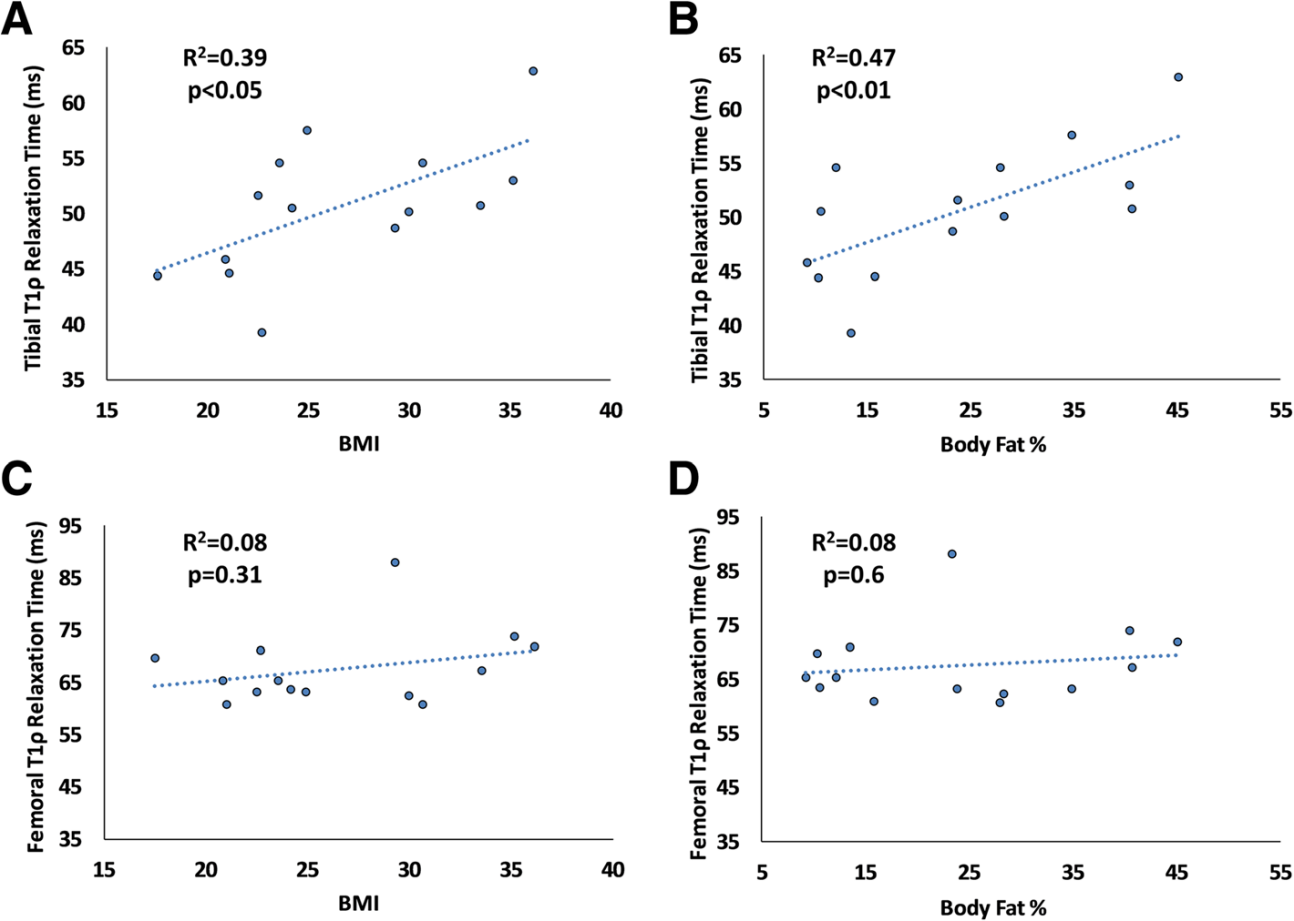
**a, b** Tibial T1rho relaxation time was significantly correlated with body mass index (BMI) (*P* <0.05) and body fat percentage (*P* <0.01). **c, d** Femoral T1rho relaxation time was not significantly correlated with BMI (*P* = 0.31) or body fat percentage (*P* = 0.6)

Pre-exercise tibial T1rho relaxation times were significantly correlated with BMI (*P* <0.05, R^2^ = 0.39, Fig. 6a), and high BMI subjects had 13% greater T1rho relaxation times compared with normal BMI subjects. Likewise, pre-exercise tibial T1rho was significantly correlated with body fat percentage (*P* <0.01, R^2^ = 0.47, Fig. 6b). However, pre-exercise femoral T1rho relaxation times were not correlated with BMI (*P* = 0.31, R^2^ = 0.08) or body fat percentage (*P* = 0.6, R^2^ = 0.08, Fig. 6c, d).

## Discussion

Obesity is a major risk factor for the development of knee OA; however, the relative contribution of biochemical and biomechanical factors to the obesity–OA relationship is unclear. In order to better understand the mechanisms by which obesity leads to OA, this study aimed to quantify both biomechanical and biochemical changes in tibiofemoral articular cartilage that occur with high BMI and increased body fat percentage. Specifically, this study explored the influence of obesity on changes in both *in vivo* cartilage strain in response to an acute, dynamic loading task (20-min treadmill walk) as well as baseline cartilage proteoglycan content using T1rho relaxation imaging. Our study found that obese subjects, who had no history of joint injury or surgery and no symptoms of knee OA, exhibited lower pre-exercise tibial cartilage thickness as well as greater tibiofemoral cartilage compressive strain following loading. Additionally, we found that high BMI subjects exhibited increased tibiofemoral T1rho relaxation times, which may be indicative of decreased proteoglycan content within the cartilage [22, 31, 32].

Increased tibial and femoral *in vivo* cartilage strains demonstrated with increased BMI are consistent with previous work from our lab which investigated diurnal changes in cartilage thickness [18]. Widmyer et al. found that over the course of normal daily activities, subjects with a high BMI (25–31) had significantly higher compressive strains in the tibial cartilage compared with those with a normal BMI (18.5–24.9) [18]. Whereas Widmyer et al. compared cartilage thickness in the morning and evening to obtain measures of diurnal strain, the current study demonstrated similar changes in tibial and femoral cartilage thickness between BMI groups as a result of a controlled and shorter duration loading activity. Specifically, this study investigated cartilage strains in response to treadmill walking for 20 min at a speed normalized to each subject’s lower limb length by using the Froude number (Fr) [26]. Controlling for walking speed across subjects by using the Froude number was important in this study as previous work has shown that given the option of self-selected walking speed, obese individuals walk slower than lean individuals, thus possibly influencing ground reaction forces and subsequent loads experienced by the knee joint [33].

Gait analysis studies have been previously used to approximate joint reaction forces in response to obesity [34–36]. For example, Harding et al. demonstrated that high BMI is associated with higher absolute tibiofemoral compressive forces by using a sagittal plane contact force model [34]. In a separate weight loss study, DeVita et al. demonstrated that weight loss of approximately 34% of initial body weight reduced maximum compressive forces in the knee as measured through gait analysis techniques, but these changes were later attenuated by gait adaptations following weight loss [35]. Additionally, Liukkonen et al. showed that weight loss induced by bariatric surgery altered knee kinetics and kinematics during gait [36]. In general, such gait analysis studies provide a wealth of information regarding knee joint loading in various populations. The present study further suggests that, in addition to potentially altering joint loading, obesity may be associated with presymptomatic alterations in the mechanical response and composition of cartilage.

In addition to changes in the mechanical function of the cartilage, we observed alterations in cartilage biochemical composition occurring with obesity. Specifically, we observed decreased proteoglycan content in the tibiofemoral cartilage of high BMI subjects as measured using T1rho imaging. Consistent with these findings, one previous study used delayed gadolinium-enhanced magnetic resonance imaging of cartilage (dGEMRIC) to estimate proteoglycan content in the articular cartilage of subjects before and after a weight loss program [37]. They concluded that weight loss was associated with protective effects on proteoglycan content and cartilage thickness [37]. Changes in cartilage composition have important implications for the mechanical behavior of cartilage and thus the development of OA [22, 31]. Specifically, decreased proteoglycan content has been shown to be related to decreased aggregate modulus, or stiffness, of cartilage resulting in increased deformation in response to load [31, 38]. Structural modifications to cartilage components such as proteoglycan loss may directly affect the ability of cartilage to withstand and transfer load. To this point, one study found that, in a diet-induced mouse model, high fat gain was associated with cartilage proteoglycan loss and reduced the aggregate modulus of cartilage in high fat–fed mice [39]. These results demonstrate that a high-fat diet may alter the material properties of cartilage by reducing proteoglycan content and decreasing tissue stiffness, potentially leading to increased deformation due to mechanical loading as observed in the present study. Importantly, chondrocyte metabolism is closely related to its mechanical environment [40], and several studies have shown that hyperphysiologic magnitudes of cartilage loading can lead to decreased synthesis of extracellular matrix components, increased production of pro-inflammatory cytokines, and potentially cell death [41–44]. Thus, altered mechanical properties related to changes in biochemical composition can change the mechanical environment experienced by chondrocytes, thus changing their metabolic activity and potentially contributing to a progressive cycle of degeneration [45]. Further support for this hypothesis is provided by evidence of decreased pre-exercise tibial cartilage thickness observed in obese subjects in this study, which may be indicative of cartilage degeneration.

The exact mechanism of obesity-related OA is unclear but may be the result of alterations in both the local mechanical loading and inflammatory environments. Specifically, it is possible that local mechanical factors, such as increased joint loading, exacerbate the effects of inflammatory cytokines (either systemic or localized), thus furthering the cycle of cartilage degeneration. Additionally, obesity is considered a systemic inflammatory disease which has been shown to induce OA in non-weight-bearing joints such as the wrist and hand [10, 46]. Recent studies suggest that metabolic factors occurring with obesity alter the activity of inflammatory cytokines that are associated with OA [47, 48]. Specifically, increased pro-inflammatory biomarkers—tumor necrosis factor alpha (TNF-α), interleukin-1 beta (IL-1β), and IL-6—have been observed in obese children with no comorbidities [47] as well as in obese patients with OA [49]. However, diet and exercise can reduce these cytokine levels [49, 50]. Such increased inflammatory cytokine activity can induce chondrocyte catabolism, resulting in degenerative changes such as decreased proteoglycan content [51, 52]. Similarly, the increased tibial T1rho relaxation time (corresponding to decreased proteoglycan content) with both increasing BMI and body fat percentage observed in the present study may be due to increased activity of inflammatory cytokines [51, 52]. The stronger correlation of tibial T1rho relaxation time with body fat percentage than with BMI demonstrated in this study may be suggestive of adipose-related inflammation playing a greater role than mechanical loading in the obesity–cartilage degeneration relationship. Future studies may further investigate the relative contributions of body mass and body fat percentage on cartilage deformation and composition.

In this study, measures of cartilage strain may have been underestimated because of inadequate recovery of the cartilage prior to the pre-exercise MRI scan or because of partial recovery prior to the post-exercise MRI scan. Although subjects were asked to refrain from strenuous activity 24 h prior to and the morning of testing, and a 45-min period of supine resting time was included to allow cartilage thickness to reach its baseline unloaded state, it is possible that the cartilage was not fully recovered prior to pre-exercise imaging. Additionally, the period between completion of the walking activity and the post-exercise MRI scan was less than 4 min. Previous work has demonstrated that cartilage volume recovers to about 50% of its original volume after 45 min of being unloaded [53]. Therefore, it is possible that some cartilage recovery occurred during this time; however, there was no difference between groups either in the study tasks completed or in the time between the completion of the walking activity and the post-exercise MRI scan. Nonetheless, cartilage recovery occurring within this 4-min period may result in underestimations of cartilage strain.

## Conclusions

The present study demonstrates a significant relationship between body composition and the biomechanical and biochemical properties of cartilage. We found that a short treadmill walking task resulted in greater tibiofemoral cartilage strains in subjects with a high BMI as compared with subjects with a normal BMI. Additionally, we found that tibiofemoral T1rho relaxation times increased in high BMI subjects, indicative of a decrease in cartilage proteoglycan concentration. Taken together with our previous work [18], these data indicate that the changes in cartilage strain observed here may be the result of alterations in both biomechanics (that is, increased joint load) and cartilage composition (loss of proteoglycan) leading to changes in mechanical properties. Importantly, decreases in proteoglycan content and cartilage thickness in obese subjects may be indicative of a “pre-osteoarthritic” state of cartilage. Characterizing the effects of BMI and body fat percentage on *in vivo* cartilage properties is a critical first step in understanding the mechanisms by which obesity alters the mechanical and biochemical properties of cartilage, thus contributing to the initiation and progression of knee OA.

## Abbreviations

3D: Three-dimensional
ANOVA: Analysis of variance
BMI: Body mass index
DESS: Double-echo steady-state
FOV: Field of view
Fr: Froude number
IL: Interleukin
MRI: Magnetic resonance imaging
OA: Osteoarthritis
TE: Echo time
TNF: Tumor necrosis factor alpha
TR: Repetition time
TSL: Spin lock time
